# Evaluation of Pain During Hysteroscopy Under Local Anesthesia, Including the Stages of the Procedure

**DOI:** 10.3390/jcm13237030

**Published:** 2024-11-21

**Authors:** Adrian Nowak, Karolina Chmaj-Wierzchowska, Agnieszka Lach, Adam Malinger, Maciej Wilczak

**Affiliations:** Department of Maternal and Child Health and Minimally Invasive Surgery, Poznan University of Medical Sciences, 60-701 Poznan, Poland

**Keywords:** mini hysteroscopy, pain, visual analog scale of pain (VAS)

## Abstract

Hysteroscopy is an endoscopic diagnostic and therapeutic method traditionally performed under general anesthesia but increasingly under local anesthesia. Today, it is considered the gold standard in gynecology. This minimally invasive procedure allows for a detailed assessment of the uterine cavity’s interior and the removal of abnormal changes within it and is applicable to patients of all ages. **Background/Objectives**: The purpose of the present study was to evaluate pain during hysteroscopy under local anesthesia at different stages of the procedure, identifying which stage is the most painful (GUBBINI Mini Hystero-Resectoscope; Tontarra Medizintechnik, Tuttlingen, Germany). **Methods**: The study included patients between the ages of 21 and 80 years. They were divided into two groups: the diagnostic hysteroscopy (HD) and the operative hysteroscopy (HO) groups. Pain measurements on the VAS scale were taken at each stage of the hysteroscopic procedure. After each stage, the operator asked the patient to indicate the maximum perceived pain value: after pericervical anesthesia was administered (VAS1), during the installation of equipment (between the removal of the speculum and the insertion of the hysteroscope into the vagina) (VAS2), after insertion of the hysteroscope and visualization of the external orifice of the cervical canal (VAS3), after passage of the hysteroscope through the cervical canal (VAS4), and after completion of the procedure in the uterine cavity (VAS5). The duration of each stage of the procedure was measured with a stopwatch: administration of pericervical anesthesia (T1), time between the removal of the speculum and the insertion of the hysteroscope into the vagina (T2), insertion of the hysteroscope into the vagina until the outer orifice of the cervical canal became visible (T3), passage of the hysteroscope through the cervical canal (T4), and the hysteroscopy procedure itself (T5). **Results**: The highest pain rating was for the canal passage stage (VAS4: 2.47 ± 2.48 points), followed by the procedure itself (VAS5: 2.12 ± 2.33 points). Anesthesia was also reported as quite painful, while the lowest pain was noted during the assembly stage. Overall pain scores for the entire procedure (VAS_max_) ranged from 3.5 ± 2.37. **Conclusion**: In conclusion, we found that the passage through the cervical canal was the most painful moment. Overall, hysteroscopy under pericervical anesthesia was not associated with significant pain. Special attention should be given to postmenopausal patients, as they experience more pain during the passage of the hysteroscope through the cervical canal. This group may benefit from additional pain management strategies during the procedure.

## 1. Introduction

Hysteroscopy is an endoscopic diagnostic and therapeutic method, traditionally performed under general anesthesia but increasingly under local anesthesia. Today, it is considered the gold standard in gynecology. This minimally invasive procedure allows for a detailed assessment of the uterine cavity’s interior and the removal of abnormal changes within it, and is applicable to patients of all ages. It is used in diagnosing and treating infertility, abnormal uterine bleeding, submucosal myomas, or endometrial polyps [1,2,3,4]. Hysteroscopy is a safe and effective procedure, with a complication risk of 0–1.5% [5,6,7] and a success rate of 94.8% [8].

The hysteroscopy procedure, performed using an endoscope with a diameter of 10 mm or larger, requires the use of a vaginal speculum, bullet forceps, and Hegar dilators to dilate the cervix [9]. The use of such instruments often causes significant pain, necessitating general anesthesia [9,10]. However, the procedure has become more common and less painful with the development and miniaturization of hysteroscopic instruments over the years. Procedures performed with 5–6 mm diameter hysteroscopes are called mini hysteroscopies [11]. Using resectoscopes with reduced diameters (e.g., the Gubbini System) eliminates the need to dilate the cervical canal, thus removing the requirement for short-term general anesthesia [12,13]. The reduction in anesthesia-associated risks, the ability to perform the procedure on an outpatient basis, and the decreased overall cost are clear advantages of this approach [14,15,16].

Despite the miniaturization of hysteroscopes, improvements in surgical technique, and the use of local anesthesia, hysteroscopy is sometimes described by patients as a painful procedure [17]. Increased pain and the risk of vasovagal reactions may lead to the procedure being abandoned [9,18]. According to the international literature, pain prevents the procedure from being completed in 1.3–5.2% of cases [19].

Discomfort during the procedure can arise from various factors. It is noted that the perception of pain and discomfort may be influenced not only by the size of the surgical instruments but also by the operator’s experience, the duration of the procedure, the patient’s psychological profile, reproductive organ abnormalities, and whether the patient has previously given birth naturally [1]. Since hysteroscopy is often performed on an outpatient basis, minimizing pain and discomfort is crucial to ensure patient comfort, which in turn affects satisfaction with the procedure. Therefore, the best pharmacological strategies and techniques to reduce pain during hysteroscopy are being actively explored.

Despite numerous studies on this topic, the evaluation of pain typically focuses on the procedure as a whole. There is a lack of studies assessing pain at the individual stages of hysteroscopy. The following stages of the procedure can be identified:

- Insertion of a speculum to visualize the cervix and administer pericervical anesthesia.
- Equipment assembly time—between the removal of the speculum and the insertion of the hysteroscope into the vagina.
- Vaginoscopy—insertion of the hysteroscope into the vagina and visualization of the external cervical canal.
- Passage of the hysteroscope through the cervical canal and into the uterine cavity.
- Diagnostic hysteroscopy, with possible biopsy or surgery for the removal of abnormal lesions.

The purpose of the present study was to evaluate pain during hysteroscopy under local anesthesia at different stages of the procedure, identifying which stage is the most painful.

## 2. Materials and Methods

The study included 129 patients admitted to the Center for Hysteroscopy under Local Anesthesia at the Heliodor Święcicki Gynecological and Obstetrical Clinical Hospital of the Karol Marcinkowski Medical University in Poznań, Poland, for hysteroscopic treatment under local anesthesia (pericervical, using lignocaine). The Center for Hysteroscopy is the first certified “CENTER OF EXCELLENCE” of The International Society for Gynecologic Endoscopy (ISGE) in Poland, for the years 2022–2024.

### 2.1. Procedure of Qualifying Patients for Surgery

The eligibility criteria for mini hysteroscopic surgery under local anesthesia (using lignocaine) included the presence of abnormal uterine bleeding, endometrial polyps or submucosal myomas, and endometrial hyperplasia. Patients reported good overall health, were generally balanced, had no history of sensitization to lignocaine or ketoprofen, and were either in phase I of their menstrual cycle or postmenopausal. Patients were excluded from the study if, at the time of qualification, they had heavy genital tract bleeding, inflammation of the vagina and/or cervix, or if pregnancy could not be ruled out.

Patients were qualified for the procedure following a gynecological and ultrasound (USG) examination and after completing a medical history. The questionnaire included questions on the patient’s age, weight, height, past surgeries, allergies, number and types of past births, number of miscarriages, previous cervical and endometrial procedures, and overall health status.

### 2.2. Data Collecting

The study included patients between the ages of 21 and 80 years. Based on reported symptoms, ultrasound findings, and the type of procedure performed, they were divided into two groups. The diagnostic hysteroscopy (HD) group consisted of women (*n* = 51) who underwent hysteroscopic endometrial biopsy due to a history of abnormal or heavy uterine bleeding. The operative hysteroscopy (HO) group consisted of women (*n* = 78) who had ultrasound evidence of endometrial polyps or submucosal myomas.

During the procedures, patients remained conscious and were able to observe the procedure in real-time on a monitor. A visual analog scale of pain (VAS) was used to assess pain at various stages of surgery. On the 10-point VAS scale, 0 indicated no pain, and 10 indicated the maximum imaginable pain. The level of pain was classified as follows: 0 for no pain, 1–3 points (mild pain), 4–7 points (moderate pain), and 8–10 points (severe pain) [20]. The principles of the scale were explained during qualification and again before surgery. Pain measurements on the VAS scale were taken at each stage of the hysteroscopic procedure. After each stage, the operator asked the patient to indicate the maximum perceived pain value: after pericervical anesthesia was administered (VAS1), during the installation of equipment (between the removal of the speculum and the insertion of the hysteroscope into the vagina) (VAS2), after insertion of the hysteroscope and visualization of the external orifice of the cervical canal (VAS3), after passage of the hysteroscope through the cervical canal (VAS4), and after completion of the procedure in the uterine cavity (VAS5).

The duration of each stage of the procedure was measured with a stopwatch: administration of pericervical anesthesia (T1), time between the removal of the speculum and the insertion of the hysteroscope into the vagina (T2), insertion of the hysteroscope into the vagina until the outer orifice of the cervical canal became visible (T3), passage of the hysteroscope through the cervical canal (T4), and the hysteroscopy procedure itself (T5).

Thus, pain was measured at each stage of the hysteroscopic procedure, while also accounting for its duration. Labels for pain assessment (VAS) and procedure time (T) were used (Table 1).

### 2.3. Surgery Procedures

After signing the appropriate consent for the hysteroscopic procedure, 100 mg of intravenous ketoprofen was administered to each qualified patient 30 min before the start of the procedure. During the procedure, vital functions were monitored, i.e., heart rate, blood pressure, O_2_ saturation, and respiratory rate. While administering perianal anesthesia, a disposable speculum was inserted to visualize the cervix, and then 10 mL each of 0.1% lignocaine solution was applied in two insertions (the first insertion at 4 o’clock and the second at 8 o’clock) using a needle (insertion depth of about 2 mm). The hysteroscopy procedure was performed under vaginoscopy without the insertion of the speculum and bullet forceps. During the operation, the patient had the opportunity to listen to music of her choice and/or talk to the medical staff.

The operators were persons of comparable experience in performing hysteroscopy. Patients for the procedure were placed on a “treatment table” in the position of a gynecological examination. A 0.9% NaCl solution with a continuous flow and a pressure of 110–120 mm Hg was used as a dilating agent for the uterine cavity. The procedure was performed using a mini hystero-resectoscope (GUBBINI Mini Hystero-Resectoscope; Tontarra Medizintechnik, Tuttlingen, Germany) with a diameter of about 6 mm. During diagnostic hysteroscopy, three endometrial microbiopsies of up to 0.5 mL each were taken, while the lesions removed during operative hysteroscopy were 0.5–1.5 cm in diameter.

### 2.4. Statistical Analysis

TIBCO Software Inc. (2017), Statistica (data analysis software system), version 13 (TIBCO Software, Palo Alto, CA, USA) and Microsoft Excel (version 2019)—Microsoft Office (Redmond, DC, USA) were used for data analysis. The distribution of variables was assessed using the Shapiro–Wilk test. Relationships between variables were examined using Kendall’s tau test. Comparisons between groups were made using the Mann–Whitney U test. A significance level of *p* < 0.05 was assumed for all calculations.

## 3. Results

### 3.1. Group Characteristics

The age of the subjects ranged from 21 to 80 years, with an average age of 43 years (45.8 ± 13.08). The BMI of the subjects ranged from 17.18 to 47.61 kg/m^2^, with an average BMI of 24.8 kg/m^2^ (26.32 ± 5.47). The obstetric history of the subjects is presented in Table 2.

### 3.2. Pain

Table 3 shows the pain scores at each stage of the procedure. The highest pain rating was for the canal passage stage (VAS4: 2.47 ± 2.48 points), followed by the procedure itself (VAS5: 2.12 ± 2.33 points). Anesthesia was also reported as quite painful, while the lowest pain was noted during the assembly stage. Overall pain scores for the entire procedure (VAS_max_) ranged from 3.5 ± 2.37. The procedure was not interrupted due to severe pain. There were no vasovagal symptoms during hysteroscopy.

Table 4 shows Kendall’s tau correlation coefficient between pain scores, anthropometric measurements, and obstetric history. The study observed that pain scores for the anesthesia stage decreased with the subjects’ age, while pain increased for the vaginoscopy stage. Similarly, for BMI, pain during the vaginoscopy stage increased with higher BMI. As the number of pregnancies, total deliveries, and natural deliveries increased, pain scores for the assembly and vaginoscopy stages also increased.

The study noted a trend toward increased pain during anesthesia in patients over 45 years of age (perimenopausal patients), although this difference did not reach statistical significance. A statistically significant increase in pain during vaginoscopy was observed in patients over 45 years of age, though this change remains clinically insignificant. No statistically significant differences were observed in pain at other stages of the procedure (Table 5). By dividing the subjects into two age groups, premenopausal and peri-/postmenopausal patients could be distinguished.

The study observed that subjects with excessive body weight had significantly higher pain scores during the vaginoscopy stage (Table 6). However, this increase was clinically insignificant. No correlation was found between the subjects’ weight and pain scores at other stages of the procedure.

Additionally, the study found no correlation between pain scores and the experience of childbirth (PSN + CC), as shown in Table 7.

Similarly, no correlation was observed between pain scores and having a natural childbirth (Table 8).

### 3.3. Procedure Time

Table 9 shows the procedure times for the various stages in minutes. The surgery stage was the longest, while the vaginoscopy stage was the shortest.

Table 10 presents Kendall’s tau correlation coefficient between procedure time, anthropometric measurements, and obstetric history. The study observed that the time required for the canal passage stage and the overall procedure time increased with the age of the subjects. However, as BMI increased, the time for the canal passage stage decreased. No correlation was found between the number of pregnancies, total deliveries, natural deliveries, and procedure time.

In the study, it was observed that the time for the canal passage stage and the overall procedure time increased with the age of the subjects (Table 11).

No effect of the patient’s BMI on the duration of the various stages of the procedure was observed (Table 12).

Additionally, no correlation was found between the delivery method (NB + CC) and the procedure time (Table 13).

There was also no correlation between the number of natural births and the duration of the procedure (Table 14).

Furthermore, no correlation was observed between the type of procedure and the duration of the other treatment stages (Table 15).

### 3.4. Evaluation of Pain Complaints Considering the Duration of the Hysteroscopic Procedure

Table 16 shows Kendall’s tau correlation coefficient between pain scores and procedure time. The study observed that a longer duration of the canal passage stage was associated with higher pain ratings for that stage. Similarly, a longer procedure stage was related to higher pain ratings for that stage. As the overall procedure time increased, both the pain rating for the procedure stage and the maximum pain index also increased.

## 4. Discussion

Currently, hysteroscopy is a recognized method for evaluating lesions of the cervical canal and uterine cavity in premenopausal and postmenopausal patients. One of the factors contributing to its popularization is the miniaturization of surgical equipment, enabling the procedure to be performed under local anesthesia on an outpatient basis. This advancement has minimized the burden on patients and improved resource management in healthcare facilities [21].

In recent years, many centers have conducted studies to evaluate and promote hysteroscopy under local anesthesia. Felix et al. highlighted the challenges faced by modern healthcare during the COVID-19 pandemic, which revealed the fragility of healthcare systems. Mini hysteroscopy allowed hospitals to admit more patients, reduce medical staff (especially anesthesiologists), and even shift procedures out of the operating room [22].

Pain and the fear of pain may deter patients from undergoing this type of procedure [23]. A study by Cicinelli and Neis suggests that hysteroscopy can be feasible and result in low VAS pain scores if patients are properly qualified [9,22]. In our previous studies, we evaluated factors such as anxiety levels and the need for information before hysteroscopy under local anesthesia (without an anesthesiologist) compared to hysteroscopy under general, intravenous anesthesia (with an anesthesiologist). We found no significant differences in anxiety levels between the two groups. However, higher pre-surgery anxiety levels were associated with longer surgery times, which in turn may increase the level of pain experienced by patients. This suggests that interventions to reduce patient anxiety are essential [24].

Additional pain-relieving techniques, such as the preoperative use of NSAIDs, as well as nonpharmacological approaches, such as anxiety management through conversation or relaxation with music, may help reduce anxiety, stress, and thus perceived pain during surgery [16,23]. However, there remains a lack of data on identifying the most painful moment of the procedure.

In the study, the anesthesia stage (VAS1) was rated as mild pain, with a mean score of 1.83 ± 1.7. This mild pain intensity is worth communicating to patients before surgery. Similar results were reported by Mody, who used a pericervical block with 20 mL of 1% lignocaine [25]. In our study, there was a tendency toward lower pain values at the anesthesia stage as the subjects’ age increased, though this did not reach statistical significance. The equipment assembly stage (VAS2) and the vaginoscopy stage (VAS3) were pain-free or associated with very mild pain, likely due to minimal contact between the instruments and the patient during these stages.

Interestingly, no significant differences were observed in pain complaints based on childbirth history. Logic dictates that one would expect less pain; however, our data and those from other centers are not homogeneous in this regard. In a study by Rolim et al., it was found that the mean VAS score during mini hysteroscopy decreased during childbirth [26], while Litt et al. found no such relationship [27].

In our study, patients over the age of 45 had significantly longer procedure times during the canal passage stage and overall. This may be attributed to age-related declines in estrogen levels, which cause vaginal mucosal atrophy, potentially leading to increased soreness during procedures such as hysteroscopy. Menopause is also associated with a reduction in uterine size, resulting in a narrower cervical canal. Chiofalo et al. observed a similar relationship, identifying cervical stenosis and menopausal status as risk factors for pain during both diagnostic and surgical stages [28]. Campas et al. also reported that the average age of patients for whom the procedure failed due to pain was 47.3 years, while the average age of those for whom the procedure was successful was 38.6 years [29]. However, these findings contradict a Hungarian study that found no differences in pain severity based on menopausal status [30].

The correlation between pain and procedure duration observed in our study can be interpreted in two ways: patients may report more pain as the procedure lengthens, or the procedure may take longer due to more challenging conditions in the cervical canal, which results in greater pain. Both mechanisms are likely to be clinically relevant. The overall pain score during the entire procedure (VAS_max_) was 3.5 ± 2.37, which is comparable to our previous study where the mean pain intensity during diagnostic and operative hysteroscopy under local anesthesia was rated at three points (3.03 ± 2.25) [31].

During the study, no vasovagal reactions were observed. This issue is variably reported in the literature. Bellingham et al. describe reactions such as bradycardia, hypotension, nausea, fainting, and vomiting occurring during or after surgery at a rate of 1.8% [32]. Cooper, in his meta-analysis, indicates that vasovagal reactions can occur in up to 20% of cases due to parasympathetic nerve irritation from surgical instruments. This issue can be mitigated by blocking the nerves with local anesthesia, significantly reducing the likelihood of vasovagal reactions [33].

Most researchers who evaluate pain during hysteroscopy focus on the time of the procedure itself. In our study, we found mild pain levels, particularly comparable to those during anesthesia and cervical canal passage. These results differ from those of Chiofalo et al., who rated the procedure stage as significantly more painful [28]. However, in their study, no premedication or local anesthesia was used, highlighting the benefit of intravenous analgesics and pericervical anesthesia before the procedure.

Nonpharmacological methods of pain relief are also worth considering. Meta-analyses show that pre-procedure anxiety increases discomfort during hysteroscopy [34]. Additionally, prolonged waiting times for the procedure can heighten anxiety, which is reflected in increased pain. In contrast, listening to music during the procedure has the potential to alleviate pain [34,35].

Studies also emphasize the importance of thoroughly answering patients’ questions about medical procedures and informing them that they can stop the procedure at any time. Informed consent is crucial, and the procedure should ideally take place at the optimal time for imaging the endometrial cavity, typically between days 4 and 14 of the cycle [36]. Although there are no established standards for premedication and anesthesia in hysteroscopy, systematic reviews have shown that the use of NSAIDs significantly reduces pain during and after the procedure, making them a recommended option [37].

## 5. Conclusions

In conclusion, we found that the passage through the cervical canal was the most painful moment. Overall, hysteroscopy under pericervical anesthesia was not associated with significant pain. Special attention should be given to postmenopausal patients, as they experience more pain during the passage of the hysteroscope through the cervical canal. This group may benefit from additional pain management strategies during the procedure.

## Figures and Tables

**Table 1:** Stages of hysteroscopy.

1. Anesthesia stage	VAS1—pain after administration of pericervical anesthesia; T1—time of administration of pericervical anesthesia.
2. Assembly stage	VAS2—pain during the installation of the equipment (between the removal of the speculum and the insertion of the hysteroscope into the vagina); T2—time between removal of the speculum and insertion of the hysteroscope into the vagina.
3. Vaginoscopy stage	VAS3—pain after the hysteroscope insertion into the vagina and until the visualization of the outer orifice of the cervical canal; T3—time of insertion of the hysteroscope into the vagina and until the external orifice of the cervical canal is visualized.
4. Canal passage stage	VAS4—pain after the hysteroscope passes through the cervical canal into the uterine cavity; T4—time of passage of the hysteroscope through the cervical canal into the uterine cavity.
5. Procedure stage	VAS5—pain after the procedure is completed; T5—duration of the hysteroscopy procedure.

**Table 2:** Past obstetric history and procedures involving cervical dilation.

	Number of Pregnancies	Number of Births	Natural Childbirths	Cesarean Sections	Miscarriages
0	36 (27.91%)	39 (30.23%)	55 (42.64%)	106 (82.17%)	112 (86.82%)
1	24 (18.6%)	24 (18.6%)	20 (15.5%)	13 (10.08%)	11 (8.53%)
2	38 (29.46%)	45 (34.88%)	35 (27.13%)	10 (7.75%)	3 (2.33%)
3	21 (16.28%)	13 (10.08%)	14 (10.85%)	-	3 (2.33%)
4	7 (5.43%)	6 (4.65%)	3 (2.33%)	-	-
5	2 (1.55%)	2 (1.55%)	2 (1.55%)	-	-
7	1 (0.78%)	-	-	-	-

**Table 3:** Pain assessment.

	N	M ± SD	Min–Max	Me [Q1–Q3]
VAS1	129	1.83 ± 1.7	0–7	2 [0–3]
VAS2	129	0.01 ± 0.09	0–1	0 [0–0]
VAS3	129	0.07 ± 0.42	0–3	0 [0–0]
VAS4	129	2.47 ± 2.48	0–10	2 [0–4]
VAS5	129	2.12 ± 2.33	0–10	2 [0–3]
VAS*_max_*	129	3.5 ± 2.37	0–10	3 [2–5]

1. Anesthesia stage (VAS1—pain after administration of pericervical anesthesia); 2. Assembly stage (VAS2—pain during the installation of the equipment—between the removal of the speculum and the insertion of the hysteroscope into the vagina); 3. Vaginoscopy stage (VAS3—pain after the hysteroscope insertion into the vagina and until the visualization of the outer orifice of the cervical canal); 4. Canal passage stage (VAS4—pain after the hysteroscope passes through the cervical canal into the uterine cavity); 5. Procedure stage (VAS5—pain after the procedure is completed).

**Table 4:** Kendall’s tau correlation coefficient between pain scores and selected factors.

	Age	BMI	Number of Pregnancies	Number of Births	Natural Births
VAS1	−0.14 *	0.05	0.01	0.02	−0.03
VAS2	0.11	0.06	0.13 *	0.13 *	0.14 *
VAS3	0.2 ***	0.16 **	0.13 *	0.14 *	0.16 **
VAS4	0.07	−0.08	−0.06	−0.08	−0.06
VAS5	0.07	0.07	0.09	0.02	0.07
VAS_max_	0.07	−0.02	0.07	0.01	0.07

1. Anesthesia stage (VAS1—pain after administration of pericervical anesthesia); 2. Assembly stage (VAS2—pain during the installation of the equipment—between the removal of the speculum and the insertion of the hysteroscope into the vagina); 3. Vaginoscopy stage (VAS3—pain after the hysteroscope insertion into the vagina and until the visualization of the outer orifice of the cervical canal); 4. Canal passage stage (VAS4—pain after the hysteroscope passes through the cervical canal into the uterine cavity); 5. Procedure stage (VAS5—pain after the procedure is completed). * *p* < 0.05; ** *p* < 0.01; *** *p* < 0.001.

**Table 5:** Age and pain assessment.

	Age						*U*	*p*
	<45 Years			>45 Years				
	M ± SD	Min–Max	Me [Q1–Q3]	M ± SD	Min–Max	Me [Q1–Q3]		
VAS1	2.04 ± 1.67	0–7	2 [1–3]	1.58 ± 1.7	0–6	1 [0–3]	1686.5	0.07
VAS2	0 ± 0	0–0	0 [0–0]	0.02 ± 0.13	0–1	0 [0–0]	2030	0.28
VAS3	0 ± 0	0–0	0 [0–0]	0.15 ± 0.61	0–3	0 [0–0]	1925	0.03
VAS4	2.2 ± 2.44	0–10	2 [0–3]	2.8 ± 2.5	0–10	3 [0–4]	1739.5	0.12
VAS5	1.93 ± 2.07	0–9	2 [0–3]	2.36 ± 2.6	0–10	2 [0–3]	1909.5	0.45
VAS_max_	3.21 ± 2.2	0–10	3 [2–5]	3.83 ± 2.53	0–10	3 [2–5]	1779	0.17

1. Anesthesia stage (VAS1—pain after administration of pericervical anesthesia); 2. Assembly stage (VAS2—pain during the installation of the equipment—between the removal of the speculum and the insertion of the hysteroscope into the vagina); 3. Vaginoscopy stage (VAS3—pain after the hysteroscope insertion into the vagina and until the visualization of the outer orifice of the cervical canal); 4. Canal passage stage (VAS4—pain after the hysteroscope passes through the cervical canal into the uterine cavity); 5. Procedure stage (VAS5—pain after the procedure is completed).

**Table 6:** Weight assessment and pain assessment.

	Body Weight Score						*U*	*p*
	Normal			Excessive				
	M ± SD	Min–Max	Me [Q1–Q3]	M ± SD	Min–Max	Me [Q1–Q3]		
VAS1	1.57 ± 1.63	0–7	1 [0–3]	2.03 ± 1.73	0–6	2 [1–3]	1697.5	0.12
VAS2	0 ± 0	0–0	0 [0–0]	0.02 ± 0.13	0–1	0 [0–0]	1982.5	0.31
VAS3	0 ± 0	0–0	0 [0–0]	0.15 ± 0.6	0–3	0 [0–0]	1885	0.04
VAS4	2.75 ± 2.67	0–10	2 [0–4]	2.19 ± 2.28	0–8	2 [0–4]	1791	0.27
VAS5	1.98 ± 2.34	0–10	1 [0–3]	2.27 ± 2.36	0–10	2 [0–3]	1837	0.38
VAS_max_	3.43 ± 2.5	0–10	3 [2–5]	3.55 ± 2.27	0–10	3 [2–5]	1918.5	0.64

1. Anesthesia stage (VAS1—pain after administration of pericervical anesthesia); 2. Assembly stage (VAS2—pain during the installation of the equipment—between the removal of the speculum and the insertion of the hysteroscope into the vagina); 3. Vaginoscopy stage (VAS3—pain after the hysteroscope insertion into the vagina and until the visualization of the outer orifice of the cervical canal); 4. Canal passage stage (VAS4—pain after the hysteroscope passes through the cervical canal into the uterine cavity); 5. Procedure stage (VAS5—pain after the procedure is completed).

**Table 7:** Childbirth experience and pain assessment.

	Childbirth Experience						*U*	*p*
	Yes			No				
	M ± SD	Min–Max	Me [Q1–Q3]	M ± SD	Min–Max	Me [Q1–Q3]		
VAS1	1.87 ± 1.81	0–7	1 [0–3]	1.74 ± 1.43	0–6	2 [0–3]	1741.5	0.95
VAS2	0.01 ± 0.11	0–1	0 [0–0]	0 ± 0	0–0	0 [0–0]	1735.5	0.52
VAS3	0.07 ± 0.39	0–3	0 [0–0]	0.08 ± 0.48	0–3	0 [0–0]	1742.5	0.84
VAS4	2.23 ± 2.31	0–9	2 [0–4]	3.03 ± 2.77	0–10	3 [0–5]	1462.5	0.13
VAS5	2.12 ± 2.37	0–10	1.5 [0–3]	2.13 ± 2.26	0–9	2 [0–4]	1748	0.97
VAS_max_	3.43 ± 2.32	0–10	3 [2–5]	3.64 ± 2.5	0–10	3 [2–5]	1678.5	0.69

1. Anesthesia stage (VAS1—pain after administration of pericervical anesthesia); 2. Assembly stage (VAS2—pain during the installation of the equipment—between the removal of the speculum and the insertion of the hysteroscope into the vagina); 3. Vaginoscopy stage (VAS3—pain after the hysteroscope insertion into the vagina and until the visualization of the outer orifice of the cervical canal); 4. Canal passage stage (VAS4—pain after the hysteroscope passes through the cervical canal into the uterine cavity); 5. Procedure stage (VAS5—pain after the procedure is completed).

**Table 8:** Natural childbirth and pain assessment.

	Natural Childbirth						*U*	*p*
	Yes			No				
	M ± SD	Min–Max	Me [Q1–Q3]	M ± SD	Min–Max	Me [Q1–Q3]		
VAS1	1.82 ± 1.84	0–7	1 [0–3]	1.84 ± 1.5	0–6	2 [0–3]	1923	0.59
VAS2	0.01 ± 0.12	0–1	0 [0–0]	0 ± 0	0–0	0 [0–0]	2007.5	0.40
VAS3	0.08 ± 0.43	0–3	0 [0–0]	0.05 ± 0.4	0–3	0 [0–0]	1990.5	0.49
VAS4	2.28 ± 2.38	0–9	2 [0–4]	2.73 ± 2.6	0–10	2 [0–4]	1824.5	0.31
VAS5	2.34 ± 2.49	0–10	2 [0–3]	1.84 ± 2.08	0–9	1 [0–3]	1812.5	0.28
VAS_max_	3.64 ± 2.37	0–10	3 [2–5]	3.31 ± 2.38	0–10	3 [2–4]	1830	0.32

1. Anesthesia stage (VAS1—pain after administration of pericervical anesthesia); 2. Assembly stage (VAS2—pain during the installation of the equipment—between the removal of the speculum and the insertion of the hysteroscope into the vagina); 3. Vaginoscopy stage (VAS3—pain after the hysteroscope insertion into the vagina and until the visualization of the outer orifice of the cervical canal); 4. Canal passage stage (VAS4—pain after the hysteroscope passes through the cervical canal into the uterine cavity); 5. Procedure stage (VAS5—pain after the procedure is completed).

**Table 9:** Procedure time.

	N	M ± SD	Min–Max	Me [Q1–Q3]
T1	129	1.17 ± 0.54	0.4–3.48	1.07 [0.85–1.38]
T2	129	3 ± 0.93	0.5–6.97	2.87 [2.5–3.4]
T3	129	0.51 ± 0.48	0.08–2.95	0.35 [0.25–0.55]
T4	129	2.23 ± 3.03	0.12–21	1.13 [0.53–2.77]
T5	129	8.9 ± 6.12	0.82–32.07	7.03 [4.77–11.25]
Total time (T)	129	15.8 ± 7.64	5.53–53.7	13.03 [10.98–19.32]

1. Anesthesia stage (T1—time of administration of pericervical anesthesia); 2. Assembly stage (T2—time between removal of the speculum and insertion of the hysteroscope into the vagina); 3. Vaginoscopy stage (T3—time of insertion of the hysteroscope into the vagina and until the external orifice of the cervical canal is visualized); 4. Canal passage stage (T4—time of passage of the hysteroscope through the cervical canal into the uterine cavity); 5. Procedure stage (T5—duration of the hysteroscopy procedure). Procedure time [minutes].

**Table 10:** Kendall’s tau correlation coefficient between procedure time and selected factors.

	Age	BMI	Number of Pregnancies	Number of Births	Natural Births
T1	0	0.06	−0.01	−0.02	0
T2	0.06	0.02	0.05	0.02	0.04
T3	0.05	0.04	−0.03	−0.02	0.02
T4	0.14 *	−0.16 **	0.01	−0.03	0.01
T5	0.06	0.02	0.02	−0.01	0.03
Total	0.13 *	0.03	0.04	−0.01	0.05

1. Anesthesia stage (T1—time of administration of pericervical anesthesia); 2. Assembly stage (T2—time between removal of the speculum and insertion of the hysteroscope into the vagina); 3. Vaginoscopy stage (T3—time of insertion of the hysteroscope into the vagina and until the external orifice of the cervical canal is visualized); 4. Canal passage stage (T4—time of passage of the hysteroscope through the cervical canal into the uterine cavity); 5. Procedure stage (T5—duration of the hysteroscopy procedure). Procedure time [minutes]; * *p* < 0.05; ** *p* < 0.01.

**Table 11:** Age and procedure time.

	Age						*U*	*p*
	<45 Years			>45 Years				
	M ± SD	Min–Max	Me [Q1–Q3]	M ± SD	Min–Max	Me [Q1–Q3]		
T1	1.16 ± 0.52	0.4–3.48	1.04 [0.85–1.42]	1.19 ± 0.57	0.42–2.97	1.1 [0.8–1.38]	2016.5	0.82
T2	2.86 ± 0.77	0.63–5.05	2.83 [2.4–3.37]	3.15 ± 1.08	0.5–6.97	2.92 [2.62–3.68]	1768	0.16
T3	0.47 ± 0.41	0.08–2.5	0.33 [0.25–0.5]	0.55 ± 0.56	0.12–2.95	0.37 [0.23–0.63]	1966.5	0.64
T4	1.58 ± 1.74	0.12–8.8	1 [0.43–1.92]	3 ± 3.94	0.17–21	1.67 [0.77–3.55]	1529	0.01
T5	8.16 ± 5.67	0.82–32.07	7 [4.45–9.72]	9.78 ± 6.55	1.82–31.32	7.38 [4.95–14.18]	1772.5	0.17
Total	14.22 ± 6.06	5.53–37.03	12.62 [9.88–16.18]	17.68 ± 8.86	7.67–53.7	15.13 [11.15–22.53]	1612	0.03

1. Anesthesia stage (T1—time of administration of pericervical anesthesia); 2. Assembly stage (T2—time between removal of the speculum and insertion of the hysteroscope into the vagina); 3. Vaginoscopy stage (T3—time of insertion of the hysteroscope into the vagina and until the external orifice of the cervical canal is visualized); 4. Canal passage stage (T4—time of passage of the hysteroscope through the cervical canal into the uterine cavity); 5. Procedure stage (T5—duration of the hysteroscopy procedure). Procedure time [minutes].

**Table 12:** Body weight assessment and procedure time.

	Body Weight Assessment						*U*	*p*
	Normal			Excessive				
	M ± SD	Min–Max	Me [Q1–Q3]	M ± SD	Min–Max	Me [Q1–Q3]		
T1	1.12 ± 0.56	0.4–3.48	1 [0.78–1.35]	1.2 ± 0.48	0.42–2.93	1.09 [0.92–1.4]	1684	0.11
T2	2.9 ± 0.84	0.63–6.83	2.83 [2.42–3.22]	3.05 ± 0.99	0.5–6.97	2.92 [2.53–3.52]	1786.5	0.27
T3	0.45 ± 0.41	0.08–2.5	0.33 [0.22–0.5]	0.57 ± 0.55	0.15–2.95	0.37 [0.28–0.55]	1667	0.09
T4	2.06 ± 1.81	0.17–6.9	1.33 [0.6–2.95]	2.28 ± 3.89	0.12–21	0.93 [0.4–2.42]	1649	0.08
T5	8.23 ± 5.35	2.43–31.32	7.03 [4.93–9.72]	9.42 ± 6.69	0.82–32.07	7.08 [4.53–13.35]	1917	0.64
Total	14.76 ± 6.17	6.93–37.53	12.62 [11.02–17.35]	16.53 ± 8.46	5.53–53.7	13.81 [10.8–21.88]	1822	0.35

1. Anesthesia stage (T1—time of administration of pericervical anesthesia); 2. Assembly stage (T2—time between removal of the speculum and insertion of the hysteroscope into the vagina); 3. Vaginoscopy stage (T3—time of insertion of the hysteroscope into the vagina and until the external orifice of the cervical canal is visualized); 4. Canal passage stage (T4—time of passage of the hysteroscope through the cervical canal into the uterine cavity); 5. Procedure stage (T5—duration of the hysteroscopy procedure). Procedure time [minutes]; BMI [kg/m^2^].

**Table 13:** Childbirth experience and procedure time.

	Childbirth Experience						*U*	*p*
	Yes			No				
	M ± SD	Min–Max	Me [Q1–Q3]	M ± SD	Min–Max	Me [Q1–Q3]		
T1	1.17 ± 0.51	0.42–2.97	1.07 [0.85–1.38]	1.19 ± 0.61	0.4–3.48	1.02 [0.83–1.5]	1748.5	0.98
T2	3.04 ± 0.98	0.5–6.97	2.87 [2.53–3.38]	2.9 ± 0.82	0.63–4.53	2.88 [2.5–3.5]	1704.5	0.80
T3	0.5 ± 0.48	0.08–2.95	0.33 [0.22–0.55]	0.53 ± 0.49	0.17–2.5	0.37 [0.27–0.58]	1574.5	0.36
T4	2.31 ± 3.37	0.12–21	1.13 [0.57–3.02]	2.03 ± 2.08	0.15–8.8	1.17 [0.48–2.73]	1746	0.97
T5	8.87 ± 6.45	0.82–32.07	7.1 [4.73–10.8]	8.98 ± 5.35	2.52–25	7 [4.97–13.35]	1651.5	0.60
Total	15.88 ± 8.18	5.53–53.7	13.21 [10.07–18.65]	15.63 ± 6.29	7.9–33.93	12.62 [11.17–21.37]	1710	0.82

1. Anesthesia stage (T1—time of administration of pericervical anesthesia); 2. Assembly stage (T2—time between removal of the speculum and insertion of the hysteroscope into the vagina); 3. Vaginoscopy stage (T3—time of insertion of the hysteroscope into the vagina and until the external orifice of the cervical canal is visualized); 4. Canal passage stage (T4—time of passage of the hysteroscope through the cervical canal into the uterine cavity); 5. Procedure stage (T5—duration of the hysteroscopy procedure). Procedure time [minutes].

**Table 14:** Natural childbirth and procedure time.

	Natural Childbirth						*U*	*p*
	Yes			No				
	M ± SD	Min–Max	Me [Q1–Q3]	M ± SD	Min–Max	Me [Q1–Q3]		
T1	1.17 ± 0.5	0.42–2.97	1.07 [0.85–1.4]	1.18 ± 0.6	0.4–3.48	1.03 [0.85–1.33]	2000.5	0.87
T2	3.07 ± 0.9	1.33–6.83	2.87 [2.53–3.5]	2.9 ± 0.97	0.5–6.97	2.88 [2.5–3.37]	1887	0.48
T3	0.51 ± 0.46	0.08–2.95	0.34 [0.23–0.63]	0.51 ± 0.51	0.17–2.53	0.35 [0.27–0.52]	2027.5	0.97
T4	2.5 ± 3.61	0.12–21	1.13 [0.58–3.35]	1.86 ± 1.97	0.15–8.8	1.17 [0.48–2.33]	1912.5	0.56
T5	9.37 ± 6.84	0.82–32.07	7.21 [4.77–11.62]	8.26 ± 4.98	2.52–25	7 [4.48–10.27]	1919.5	0.58
Total	16.62 ± 8.76	5.53–53.7	14.02 [10.6–21.8]	14.71 ± 5.71	7.9–33.93	12.58 [11.02–18.45]	1854.5	0.39

1. Anesthesia stage (T1—time of administration of pericervical anesthesia); 2. Assembly stage (T2—time between removal of the speculum and insertion of the hysteroscope into the vagina); 3. Vaginoscopy stage (T3—time of insertion of the hysteroscope into the vagina and until the external orifice of the cervical canal is visualized); 4. Canal passage stage (T4—time of passage of the hysteroscope through the cervical canal into the uterine cavity); 5. Procedure stage (T5—duration of the hysteroscopy procedure). Procedure time [minutes].

**Table 15:** Type of hysteroscopy and procedure time.

	Hysteroscopy Type						*U*	*p*
	Diagnostics			Surgical				
	M ± SD	Min–Max	Me [Q1–Q3]	M ± SD	Min–Max	Me [Q1–Q3]		
T1	1.26 ± 0.57	0.42–2.97	1.13 [0.88–1.45]	1.12 ± 0.51	0.4–3.48	1 [0.83–1.35]	1648.5	0.10
T2	2.8 ± 0.7	0.5–4.48	2.82 [2.5–3.1]	3.12 ± 1.04	0.63–6.97	2.93 [2.5–3.55]	1674.5	0.13
T3	0.58 ± 0.55	0.08–2.53	0.43 [0.27–0.65]	0.46 ± 0.43	0.08–2.95	0.33 [0.23–0.5]	1600	0.06
T4	2.54 ± 3.17	0.12–19.08	1.35 [0.58–3.28]	2.02 ± 2.94	0.15–21	1 [0.5–2.43]	1717.5	0.19
T5	7.26 ± 5.08	1.82–31.32	5.97 [4.17–8.12]	9.97 ± 6.52	0.82–32.07	7.88 [4.95–13.5]	1472.5	0.01
Total	14.45 ± 6.41	7.67–37.53	12.62 [9.68–16.3]	16.69 ± 8.27	5.53–53.7	13.65 [11.17–21.88]	1671.5	0.13

1. Anesthesia stage (T1—time of administration of pericervical anesthesia); 2. Assembly stage (T2—time between removal of the speculum and insertion of the hysteroscope into the vagina); 3. Vaginoscopy stage (T3—time of insertion of the hysteroscope into the vagina and until the external orifice of the cervical canal is visualized); 4. Canal passage stage (T4—time of passage of the hysteroscope through the cervical canal into the uterine cavity); 5. Procedure stage (T5—duration of the hysteroscopy procedure). Procedure time [minutes].

**Table 16:** Kendall’s tau correlation coefficient between pain scores and procedure time.

	T_1–5_	Total Time
VAS1	−0.02	−0.04
VAS2	0.11	−0.03
VAS3	0.09	0.03
VAS4	0.4 ***	0.1
VAS5	0.15 **	0.23 ***
VAS_max_	-	0.18 **

1. Anesthesia stage (VAS1—pain after administration of pericervical anesthesia, T1—time of administration of pericervical anesthesia); 2. Assembly stage (VAS2—pain during the installation of the equipment—between the removal of the speculum and the insertion of the hysteroscope into the vagina, T2—time between removal of the speculum and insertion of the hysteroscope into the vagina); 3. Vaginoscopy stage (VAS3—pain after the hysteroscope insertion into the vagina and until the visualization of the outer orifice of the cervical canal, T3—time of insertion of the hysteroscope into the vagina and until the external orifice of the cervical canal is visualized); 4. Canal passage stage (VAS4—pain after the hysteroscope passes through the cervical canal into the uterine cavity, T4—time of passage of the hysteroscope through the cervical canal into the uterine cavity); 5. Procedure stage (VAS5—pain after the procedure is completed, T5—duration of the hysteroscopy procedure). Procedure time [minutes]. ** *p* < 0.01; *** *p* < 0.001.

## Data Availability

The data presented in this study are available on request from the corresponding author.
